# The Notable Achievements and the Prospects of Bacterial Pathogen Genomics

**DOI:** 10.3390/microorganisms10051040

**Published:** 2022-05-17

**Authors:** Grigorios D. Amoutzias, Marios Nikolaidis, Andrew Hesketh

**Affiliations:** 1 Bioinformatics Laboratory, Department of Biochemistry and Biotechnology, University of Thessaly, 41500 Larissa, Greece; marionik23@gmail.com; 2 School of Applied Sciences, University of Brighton, Huxley Building, Lewes Road, Brighton BN2 4GJ, UK; a.hesketh@brighton.ac.uk

**Keywords:** bacterial pathogen, genomics, Illumina, Pacific Biosciences, Oxford Nanopore, evolution, forensics, food-borne pathogens, clinical microbiology, metagenome-assembled genomes

## Abstract

Throughout the entirety of human history, bacterial pathogens have played an important role and even shaped the fate of civilizations. The application of genomics within the last 27 years has radically changed the way we understand the biology and evolution of these pathogens. In this review, we discuss how the short- (Illumina) and long-read (PacBio, Oxford Nanopore) sequencing technologies have shaped the discipline of bacterial pathogen genomics, in terms of fundamental research (i.e., evolution of pathogenicity), forensics, food safety, and routine clinical microbiology. We have mined and discuss some of the most prominent data/bioinformatics resources such as NCBI pathogens, PATRIC, and Pathogenwatch. Based on this mining, we present some of the most popular sequencing technologies, hybrid approaches, assemblers, and annotation pipelines. A small number of bacterial pathogens are of very high importance, and we also present the wealth of the genomic data for these species (i.e., which ones they are, the number of antimicrobial resistance genes per genome, the number of virulence factors). Finally, we discuss how this discipline will probably be transformed in the near future, especially by transitioning into metagenome-assembled genomes (MAGs), thanks to long-read sequencing.

## 1. Bacterial Pathogen Genomics: A Young Discipline Addressing an Old Problem

The first documented case of a disease that spread from an infected person to others was recorded in the city-state of Athens by the Greek historian Thucydides during the Peloponnesian War between 430–426 B.C. More than a quarter of the population died in an epidemic that became known as the plague of Athens, but the causative agent remains unknown to this day. Eighteen hundred years later, the Black Death killed more than a third of the European population during the 1347–1352 epidemic, with *Yersinia pestis* only much later being proposed as the bacterium responsible [1]. The heavy tolls that infectious diseases have inflicted on the human population throughout history have been a driving force behind the scientific efforts to understand and control them, but it was not until recently, in 1876, that a specific microorganism was attributed to a particular disease, with the identification of *Bacillus anthracis* as the etiological agent of anthrax by Robert Koch [2]. The works by Koch and Luis Pasteur during that period were instrumental in establishing the germ theory of disease. Almost fifty years later, in 1928, Alexander Fleming made a discovery that later led to the development of the first antibiotic, penicillin. This opened up an era of antimicrobial drug discovery, driven both by the promise of defeating bacterial pathogens that had long had a negative impact on human health and well-being, and by the realisation that specific antibiotics could have limited usefulness due to the development and spread of resistance in bacterial populations. Just 17 years after the introduction of penicillin, the first clinical case of penicillin-resistant *Staphylococcus aureus* was reported [3].

The era of microbial genomics can be viewed as starting in 1992 with the sequencing of an entire chromosome, chromosome III, of the unicellular eukaryotic microbe *Saccharomyces cerevisiae* [4]. This was quickly followed in 1995 by the publication of the first two complete bacterial genomes by Craig Venter and colleagues, those of the opportunistic human pathogens *Haemophilus influenza* and *Mycoplasma genitalium* [5,6]. A remarkable finding from the 0.57 Kbp genome of *M. genitalium* was that cellular life can be based on less than 500 genes [7]. Only one year later, the first unicellular eukaryotic genome, that of the yeast *S. cerevisiae,* was also sequenced [8] and since then the data on microbial genomes and the functions they encode have been continually expanding (for a review see [9]). In 2010, the first self-replicating bacterial cell of *Mycoplasma mycoides* JCVI-syn1.0, with a synthetic genome of 1.08 Mbp was designed, thus heralding the era of genome engineering [10]. Nowadays, hypothesis-driven science is complemented by data-driven (inductive) science [11].

In the sections below, we present an overview of the current status of bacterial pathogen genomics, outline some of the technological advances that have enabled its growth and development, and consider the ways in which it is increasingly impacting the understanding, control, and treatment of pathogenic human diseases.

## 2. A Relatively Small Number of Pathogenic Bacteria Are of High Importance and Impact

More than one thousand different bacteria can be pathogenic for humans; however, the vast majority of cases reported in the clinical setting comprise a very small sub-set. A list of the top 25 bacteria most frequently identified by a microbiology laboratory was compiled by [12], where *Escherichia coli* and *S. aureus* comprise almost half of the cases, followed by *Enterococcus faecalis*, Coliforms, *Streptococcus*, *Pseudomonas*, etc. Accordingly, a recent nationwide study in Denmark compared classical species identification with that by whole-genome sequencing (WGS) in the clinical setting. They also identified *E. coli* and *Staphylococcus* spp. as the two most clinically prevalent bacteria [13]. In addition, the emerging problem of antibiotic-resistant strains forced the World Health Organisation to publish a priority list of 12 bacterial taxa for which new antibiotics need to be researched and developed [14]. The list contains three priority sub-categories, with *Acinetobacter baumanii*, *Pseudomonas aeruginosa,* and *Enterobacteriaceae* (all of them carbapenem-resistant) being designated as Priority 1 (Critical). Furthermore, concerning biosecurity and biodefence, the U.S. Federal Select Agent Program (FSAP) has issued a list of more than 15 bacteria that have been determined to have the potential to pose a severe threat to humans, and/or animals, and/or plants [15]. Seven of those bacteria (*Bacillus cereus* biovar *anthracis,* Botulinum neurotoxin producing *Clostridium*, *Francisella tularensis, Y. pestis, B. anthracis, Burkholderia mallei*, *Burkholderia pseudomallei*) have been classified as Tier 1, being the most dangerous. In addition, a survey that was conducted among plant bacteriologists identified the top 10 bacterial plant pathogens based on scientific and economic importance, with *Pseudomonas syringae* pathovars being ranked first [16]. However, there is no room for complacency since a new bacterial pathogen may emerge or re-emerge at any time.

## 3. Short-Read Sequencing Technologies Have Enabled the Systematic Study of Pathogenic Bacteria

Improvements in DNA sequencing technologies have underpinned the development and expansion of the field of pathogen genomics. Coupled with advances in computational biology, genome-wide sequence-based approaches have enabled a systematic consideration of evolutionary processes in bacteria in relation to virulence, transmission, antibiotic resistance, and susceptibility. These approaches are also being used to implement more rapid and effective methods for infection surveillance and tracking that are capable of having an impact on disease prevention and control. The emergence of cost-effective second-generation short-read DNA sequencers in 2005–2010 made it possible to sequence any bacterial genome of interest, and kick-started the accumulation of the wealth of information on the bacterial gene sequences and the proteins they encode that we see today. Initial limitations of read-length, typically 35–50 bp in the earliest short-read platforms, precluded the instant assembly of complete genome sequences. However, the step-change in sequencing capacity did allow for the rapid assembly of draft genomes, comprising relatively small numbers of very long DNA contigs together with a large number of small contigs. Sequence repeats larger than the read length were the main reason for not being able to fully assemble a bacterial chromosome and plasmids, but incomplete draft genome assemblies are still a rich source of information. They can also be further polished into completed genomes, if desired, by using a variety of gap-closing approaches. With continual improvements in sequencing length, accuracy, convenience, and affordability, Illumina platform sequencers have emerged as a dominant force in short-read sequencing. For example, a recent large-scale global study of more than 10,000 *Salmonella* genomes was achieved with Illumina sequencing, where the total cost of consumables (DNA extraction and genome sequence generation—excluding staff time) was under USD 10 per sample [17]. However, the limitations due to read length, now typically 75–300 bp, and the requirement for template sequence amplification prior to sequencing do still apply.

## 4. The Promise of Long-Read Sequencing Technologies and Hybrid Approaches

Over the last decade, third-generation long-read sequencing technologies have emerged to address the limitations referred to above, most notably from Pacific Biosciences (PacBio) [18] and Oxford Nanopore Technologies (ONT) [19]. These can achieve read length orders of magnitude higher than those produced by the short-read platforms. However, their initial error-rates were very high (10–15%) [20]. The latest PacBio Sequel IIe system generates reads of average length 10–15 kb, producing 500 Gb of data within 30 h of running [20]. The PacBio circular consensus sequencing (CCS) can now generate high fidelity (HiFi) long-reads with an error-rate of less than 1% [21]. As a comparison, the Oxford Nanopore PromethION 48 generates reads with a maximum length of more than 4 Mb, producing 14 Tb of data within 72 h of running [20]. The new ONT R.10 chemistry allows for a mean consensus single molecule error-rate of less than 1%. These two technologies can sequence non-amplified input DNA, and tend to require simpler sequencing library preparation protocols that are quicker to perform (minimum time of 10 min for library construction) than those needed for short-read sequencing (several days) [22]. Oxford Nanopore devices are now also capable of displaying the generated sequence in real-time, enabling adaptive sampling of the reads being sequenced, in order to select (or reject) reads by their sequence properties as they transit through the nanopores. In addition, Oxford Nanopore devices have the advantage of being very portable, with a proven track record even in extreme environments such as the International Space Station [23]. However, current deficiencies in both base-calling accuracy and cost-efficiency, relative to short-read platforms, have meant that third-generation long-read sequencing has not yet overtaken short-read sequencing in popularity.

**Figure 1:**
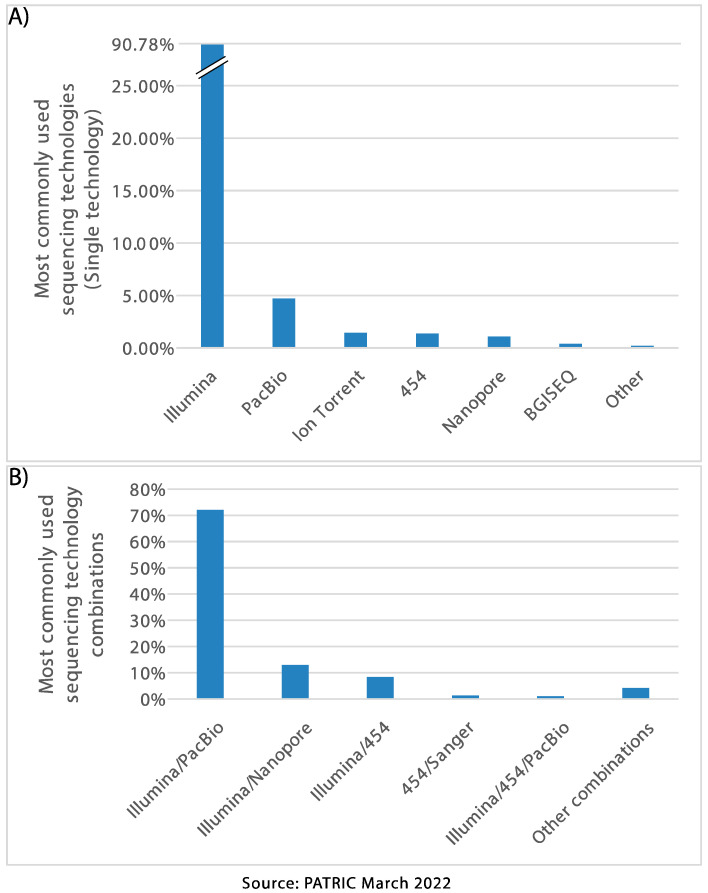
Most frequently used sequencing platforms according to PATRIC, for bacterial pathogens, (**A**) used as single technology and (**B**) used in combinations (hybrid approaches).

The relative strengths and weaknesses of the second- and third-generation sequencing technologies mean that one platform, or the other or even a hybrid approach, may be more suitable to a certain research project. Studies requiring whole-genome sequencing (WGS) of a large number of related strains can benefit from the accuracy and cost-effectiveness of short-read sequencing [17,24], but can additionally include a long-read platform to gap-close if the aim is to also produce more complete genome sequences or analyse the plasmid content [25]. According to the metadata that we mined from PATRIC (March 2022; Figure 1), the most frequently used platform when sequencing a bacterial pathogen with only one technology is Illumina (~91% of sequenced strains), followed by PacBio (5%). For hybrid assemblies (short and long reads), the most common combination is Illumina with PacBio (72% of hybrid assemblies), followed by Illumina with Oxford Nanopore (13% of hybrid assemblies). These figures are likely to change in the near future as the uptake of third-generation platforms increases.

## 5. Pathogen Genomics Bioinformatics Resources: A Wealth of Sequence Data for Comparative Genomics

The vast amount and complexity of the genomic data that are produced by diverse technologies require sophisticated data store solutions and bioinformatics tools/pipelines and analyses. Among the most important bacterial pathogen genomics resources are (i) the NCBI pathogens database [26], which is associated with the NCBI pathogen detection program; (ii) the Pathosystems Resource Integration Centre (PATRIC) [27]; (iii) Pathogenwatch at the Wellcome Trust Sanger Institute [28]; and (iv) JGI’s Integrated Microbial Genomes and Microbiomes system (IMG/M; although this is not limited to pathogens) [29].

**Figure 2:**
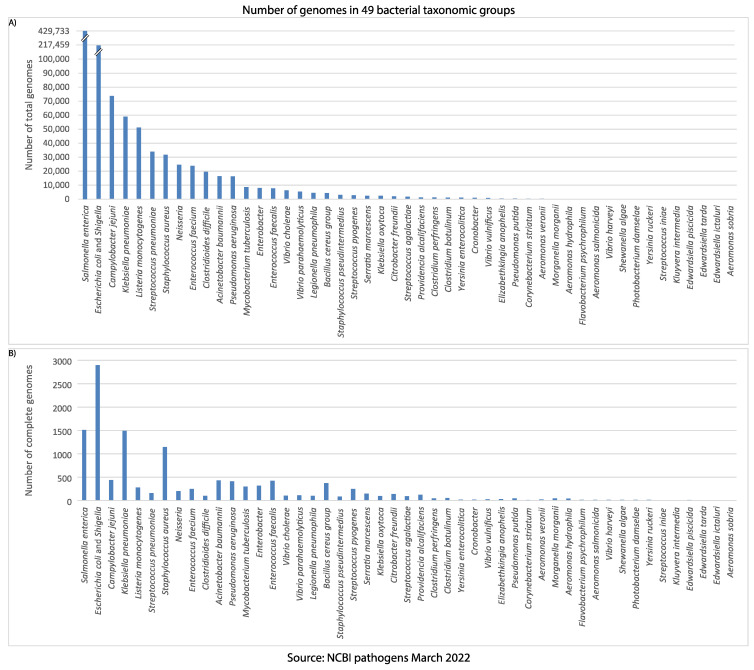
The number of genomes in each bacterial taxonomic group of the NCBI pathogens. (**A**) The total number of genomes reported in each taxonomic group. (**B**) The number of complete genomes in each taxonomic group.

The NCBI pathogens database contains the genomic sequence data of the pathogenic bacteria and fungi from food-borne, environmental, and clinical sources. In total, it contains the genome data for more than a million isolates from 49 bacterial groups (Figure 2), comprised of 33 genera, with 1.18% (12,573) of them being annotated as complete genomes. The most frequently sequenced taxon is *Salmonella enterica* (Figure 2), with more than 429,000 genome sequences, followed by the *E. coli—Shigella* group, with more than 215,000 genome sequences. Nevertheless, the *E. coli—Shigella* group has the highest number of complete genome sequences (2898). The most prominent host is human, followed by chicken.

PATRIC started as a database for the comparative genomics of bacterial pathogens and has grown to be a data-rich bioinformatics resource centre not limited to prokaryotes [27]. This database includes 548,000 bacterial genomes (mostly obtained from the NCBI Genbank and RefSeq), with almost 36,000 (7%) of them being complete. More than 250,000 genomes have curated metadata relating the pathogens to the diseases they cause. Of these, around 5800 genomes, annotated as “High Quality”, have corresponding disease information with the top three being MRSA-positive infection (methicillin-resistant *S. aureus*; ~780 genomes), shigellosis (~580 genomes), and typhoid fever (~530 genomes). In total, PATRIC reports about 500 different diseases from high-quality bacterial genome sequences. PATRIC also offers several bioinformatic workflows that are available through the web browser or the dedicated command-line interface (CLI).

Pathogenwatch [28] can quickly process genomes to perform multi-locus sequence typing (MLST), identify genes and SNPs that are implicated in antimicrobial resistance, and infer their susceptibility to antibiotics. Additionally, it provides the closest phylogenetic neighbours and their geographical location (if available). All this information is available via an interactive interface and can be downloaded. As of April 2022, Pathogenwatch contains more than 73,000 submitted bacterial genomes.

## 6. Genome Assembly

**Figure 3:**
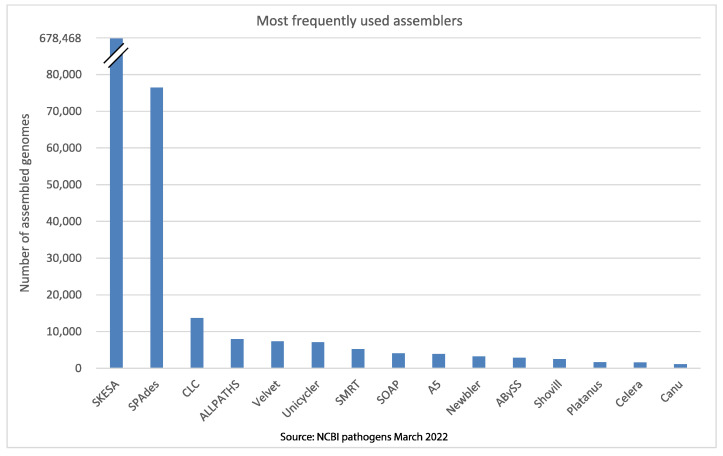
Most of the commonly used assemblers reported in the NCBI bacterial pathogens database as of March 2022.

A key step in microbial genomics is to be able to accurately assemble single genome and plasmid sequences from the thousands/millions of shorter DNA sequences produced by the platforms discussed above. An analysis of the most commonly used genome assemblers in NCBI pathogens revealed that SKESA is by far the most frequently used (678,468 total entries—82% of total genomes with available assembler metadata), followed by SPAdes (Figure 3).

SKESA is a short-read assembler designed by the NCBI staff for the de novo assembly of microbial genomes sequenced with the Illumina technology [30]. The assembler is integrated into the NCBI RAPT (Read assembly and Annotation Pipeline Tool) [31] and is utilised by the Pathogen Detection Project (PDP), which investigates food-borne disease outbreaks using NGS technologies. SPAdes is a popular assembler that can utilise short-reads or perform the assembly in a hybrid manner using both short- and long-reads from various sequencing platforms [32]. This software was initially designed to handle Illumina reads for the de novo assembly of genome sequences from the bacteria cultured in the laboratory, but now also supports IonTorrent, PacBio, and Nanopore platforms.

Since the assembly of genomes is a computationally demanding task, researchers may wish to rely on online services that provide the necessary computer power instead of using their own computational resources and bioinformatics pipelines. One such example is the NCBI RAPT pipeline, but it only allows for the use of the SKESA assembler. The PATRIC assembly service allows the user to choose between SPAdes and other assemblers and is able to handle the Illumina, Ion Torrent, PacBio and Nanopore reads. The user may also choose whether or not to polish the assembly product using other software. For more information on the PATRIC assembly service, one can also refer to the official website (https://docs.patricbrc.org/tutorial/genome_assembly/assembly.html (accessed on 13 April 2022)).

## 7. Genome Annotation—Inferring Taxonomy, Pathogenicity, and Antimicrobial Resistance

### 7.1. The RAPT, RAST, and PROKKA Annotation Pipelines

The reliable automatic annotation of genomic data is crucial when studying pathogens since it can provide information about the virulence and antimicrobial resistance. The Read assembly and Annotation Pipeline Tool (RAPT) is very popular for genome assembly, annotation, and taxonomic classification. For gene annotation, it uses the Prokaryotic Genome Annotation Pipeline (PGAP) [33]. The final product of annotation contains information about the pseudogenes, non-coding genes, coding genes including antimicrobial resistance (AMR) genes, and virulence factors. The AMR genotypes are predicted using the amrfinder tool [34]. Each genome submitted into RAPT is also taxonomically classified using the NCBI specialised average nucleotide identity (ANI) pipeline against the reference assemblies, as described in [35].

Another computational resource that performs genome annotation is the RAST toolkit (Rapid Annotation using Subsystem Technology) [36] and is integrated into PATRIC. RAST can be run online without the need for installation, but local installation is an available option through the PATRIC command line interface (CLI; https://github.com/PATRIC3/PATRIC-distribution (accessed on 13 April 2022)). This tool is highly modular and allows users to create custom pipelines based on their criteria and data. The toolkit version reported in [37] contains 16 basic modules that are used during the default pipeline. By default, the tool outputs the protein-coding genes, the non-protein-coding genes, repeat regions, predicted pyrrolysyl and selenoproteins, and CRISPR elements. The user can also utilise five more modules to extend the analysis parameters, according to the needs of the project. Such examples are the prophage-phispy and insertion-sequence modules that implement the PhiSpy algorithm for prophage detection [38] and BLAST search against the SEED and Isfinder databases [36,39], respectively. RAST also offers a module to identify the special genes that are implicated in virulence, AMR, and those that may be potential drug targets.

Prokka is another very popular tool that can rapidly annotate (within 10 min) prokaryotic genomes [40]. It is a command-line tool that can be installed locally in a Linux system.

### 7.2. Accurate Taxonomic Classification in the Genomic Era

For many years, bacterial taxonomy was based on the morphological and phenotypic characteristics that are inherently susceptible to homoplasy. The application of the 16S ribosomal RNA by Carl Woese allowed for a deeper and more accurate representation of the bacterial taxonomy [41]. However, the resolution of this single-gene method was not sufficient for accurate classification at the species and sub-species level [42]. The development of MultiLocus Sequence Typing (MLST) based on (i) a few (5–7) housekeeping genes, or (ii) 53 ribosomal genes (termed rMLST) [43], or (iii) hundreds to thousands of core-genome genes (termed cgMLST), or (iv) thousands of core-genome single nucleotide variations (termed cgSNV) [44] allowed for even higher levels of resolution (depending on the number of genes/SNPs analysed), even at the strain-level.

Bacteria may demonstrate very diverse phenotypes, even within groups of the same species. Therefore, certain well-studied pathogens have been classified (within the same species) into different serogroups and serotypes based on selected biochemical assays/phenotypes and antigen tests. This is important for predicting the pathogenic profile of the strain or for studying outbreaks. For example, by using the classical typing scheme, *Shigella* strains are classified into four serogroups and more than 50 serotypes [45]. However, a recent phylogenomic analysis identified eight distinct phylogenetic clusters. Members of the same serotype may belong to different clusters. In addition, evolutionarily related members may belong to different serotypes. The classical serotyping scheme is problematic due to horizontal gene transfer and IS element inactivation.

PubMLST is a very popular web-based bioinformatics resource that allows for the accurate taxonomic classification of bacterial pathogen sequences, even at the strain level [46]. This resource uses the BIGSdb software [47] and a collection of over 100 manually curated species-specific or genera-specific databases. It can taxonomically classify a submitted sequence, based on either the MLST, rMLST, or cgMLST methods. The PubMLST system further integrates genetic variation with provenance (time and place) and phenotypic data, in order to predict antimicrobial resistance, cross-reactivity with vaccine antigens, and other key phenotypes. Enterobase is another software environment that uses cgMLSTs or cgSNVs to identify the global population structure (at multiple levels of resolution) of several important bacterial genera such as *Escherichia*, *Salmonella*, *Yersinia*, *Clostridioides*, *Helicobacter*, *Vibrio*, and *Moraxella* [48].

Concerning species demarcation, the average nucleotide identity (ANI) method is routinely utilised. It compares pairs of whole-genome sequences with a cut-off of 95% nucleotide identity [49,50]. Several tools are available. The RAPT pipeline uses MegaBLAST to calculate the ANI values of a submitted genome against a set of reference genomes and determine its species status. Since the computation of ANI values is crucial in the era of ever-expanding genomic datasets, other software have also been developed that can be installed and run on dedicated local machines. One such example is the pyani software/module that uses either MUMMER (ANIm) or BLASTn (ANIb) as the aligner. FastANI [51] is designed to compare complete genomes by utilising the Mashmap sequence mapping algorithm instead of alignment software. This alignment-free approach is significantly faster than other similar methods, with minimal loss of accuracy.

### 7.3. Prediction of Antimicrobial Resistance Genes and Phenotypes

One major challenge in this new era of bacterial whole genome sequencing is to predict the antimicrobial resistance phenotype rapidly and accurately from the genotype. Many different molecular mechanisms may confer resistance to a certain antibiotic [52] such as (i) modification (i.e., aminoglycoside modifying enzymes, chloramphenicol acetyltransferases) or destruction of the antibiotic (i.e., beta-lactamases, AmpC enzymes and carbapenemases); (ii) reduced accumulation of the antibiotic (increased activity of efflux pumps or decreased permeability of porins); (iii) alterations at the target of the antibiotic (such as overproduction, replacement, enzymatic alterations/modifications, mutations); (iv) or even by metabolic bypassing of the target via alternative pathways. Several manually curated databases have been developed in order to mine all the genes and mutations that are known (experimentally) to confer resistance to certain antibiotics (e.g., a mutation in gyrase subunit A conferring resistance to fluoroquinolone) from the literature [53]. Based on these databases, the accompanying bioinformatics tools use homology search or identify specific known mutations in target genes, in order to predict the antimicrobial resistance from WGS data (either assembled genomes or even raw reads). Notable examples include (i) the Comprehensive Antibiotic Resistance Database (CARD) with the accompanying Resistance Gene Identified (RGI) tool [54]; (ii) the ResFinder/PointFinder databases/tools [55]; (iii) the Bacterial Antimicrobial Resistance Reference Gene Database/Catalogue and the accompanying AMRFinderPlus detection tool [34]; and (iv) the standalone Antibiotic Resistance Gene-ANNOTation (ARG-ANNOT) database/tool [56]. The main weakness of these approaches is that only well-characterised AMR mechanisms can be detected, whereas other less characterised mechanisms will be missed. In addition, all these homology-based prediction methods depend on curator-specified similarity thresholds (usually BLAST or the Hidden Markov model bit-score thresholds), which may significantly impact the predicted phenotype, if they become more or less stringent.

### 7.4. Databases and Prediction Tools for Virulence Factors

The two most popular databases for virulence factors are the Virulence Factor DataBase (VFDB) [57] and Victors [58]. VFDB is a database that has collected virulence factors with text mining and has organised them into 14 major categories (adherence, invasion, effector delivery system, motility, exotoxin, exoenzyme, immune modulation, biofilm, nutritional/metabolic factor, stress survival, post-translational modification, antimicrobial activity/competitive advantage, regulation, others). These categories are further organised into more than 100 subcategories, in a hierarchical architecture. Until recently, one major challenge in this field was the absence of a unified classification scheme for all VFs from different bacterial pathogens. Accordingly, VFDB has recently re-organised the classification scheme to tackle this problem. Another challenge is that many bacterial VFs may have more than one function. VFanalyzer is a pipeline of the VFDB that scans complete or draft genomes (provided by the user) for virulence factors based on the data from the VFDB. At first, the protein-coding genes of the query genome are predicted and then clustered into orthologous groups with the reference VFDB proteins. Proteins that are not assigned to an orthologous group are then fed to a series of homology searches using the BLAST (mostly) and Hidden Markov models against experimentally verified and predicted virulence factors with strict cut-offs. Since most of the virulence factors are organised in genomic clusters, the final predicted VF genes are validated using their genomic location to verify the cluster integrity (if possible). In addition, the VFDB displays a circular and linear pathogenomic map of a given bacterial genome when using the CGView tool [59]. Thus, pathogenicity clusters may be observed, where the locations of the various VFs are displayed, and they are coloured, according to the VF category they belong to.

The Victors database integrates the data of virulence factors by manual curation of the published literature. This database is not limited to bacterial pathogens and currently (as of April 2022) contains 4570 virulence factor sequences from 61 bacterial species, which belong to 38 genera. The sequences that are selected by curators are then processed by several bioinformatics tools to predict further information such as subcellular localisation, pathogen–host interactions, protein–protein interactions, and the COG category, if available. The genus with the most virulence factors is *Escherichia*, which has 566 sequences, followed by *Streptococcus* with 519 sequences. Furthermore, the user has the ability to BLAST query sequences against the Victors database using a dedicated web tool.

### 7.5. Identification and Prediction of Genomic Islands

The identification and analysis of horizontally transferred genomic islands (GIs) is also very important in bacterial pathogen genomics; GIs frequently encode virulence factors [60,61] (termed pathogenicity islands) and/or antimicrobial resistance genes [62,63] (termed resistance islands) or mixtures of them. Many computational tools for GI detection have been developed that use local nucleotide composition bias, the presence of mobility/hypothetical/phage-related genes, direct repeats, and insertion sequences (for an extensive review of various computational tools see [64]). In addition, there exist several databases of predicted and/or curated GIs such as IslandViewer [65], the Pathogenicity Island Database (PAIDB) [66], and the MobilomeDB/VRprofile database/webserver [67]. A recent assessment of 20 composition-based prediction systems determined that the highest precision and recall were achieved by the IslandViewer 4 composite prediction system and the GIHunter method [64].

### 7.6. Virulence and AMR Metrics in the NCBI Pathogens Database

**Figure 4:**
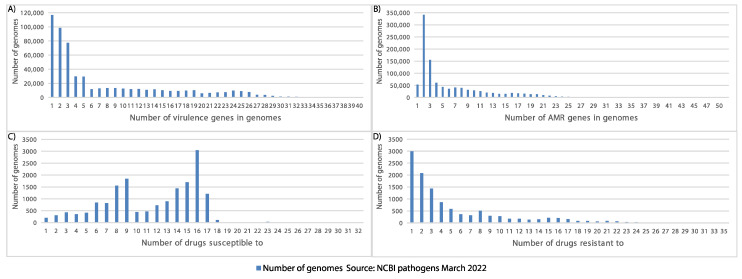
Pathogen annotation data for the virulence, resistance, and drug susceptibility. (**A**) The number of genomes with a certain number of virulence genes. (**B**) The number of genomes with a certain number of AMR (antimicrobial resistance) genes. (**C**) The number of genomes with a certain number of drugs to which they are susceptible (based on experiments). (**D**) The number of genomes with a certain number of drugs to which they are resistant (based on experiments). Source: The NCBI pathogens (March 2022).

Based on the available metadata from the NCBI pathogens, we calculated the metrics concerning various pathogenicity-related characteristics such as the number of virulence factors per genome, the number of AMR genes per genome, the number of drugs to which a sequenced strain is susceptible, and the number of drugs to which a sequenced strain is resistant. As seen in Figure 4A, most of the sequenced strains harboured one to three virulence genes. Furthermore, more than half of the strains (53%; 549,910/1,044,081) with predicted AMR genes contained two or three of them (Figure 4B). Almost half of the strains (~50%; 8412/16,977) with the available experimental data were susceptible to 1 to 12 different drugs (Figure 4C). More than half of the strains with the available experimental drug resistance data (57%; 6505/11,464) were resistant to one to three different drugs (Figure 4D).

## 8. The Contribution of Bacterial Pathogen Genomics in Fundamental Research

Within these 27 years from the first bacterial genome sequenced, an unprecedented wealth of data and knowledge has been gained about the evolution of bacteria [68,69]. A very comprehensive bacterial taxonomy is now available, based on the phylogenomics of more than 94,000 bacterial genomes [70]. Important conceptual shifts have also been achieved such as the notion of core/accessory and dynamic pangenomes [71,72]. Horizontal gene transfer and homologous recombination have emerged as major evolutionary forces in bacteria and have challenged the idea of a single tree of life [73,74,75,76,77,78]. At the same time, genomics is shedding light on the evolution of pathogenicity [79]. For example, mobile genetic elements have been found to play a key role in the transformation of the harmless commensal *E. coli* into a pathogen that can cause a wide range of diverse diseases (eight pathotypes) [80,81,82]. A SNP in the AmpC beta-lactamase of Phylogroup A *E. coli* confers resistance to third-generation cephalosporins [83]. Another commensal bacterium, *S. aureus*, may be transformed into a pathogenic form that causes severe infections via the adaptive evolution of quorum sensing, surface antigen, or toxin-producing genes [84]. Recently, there has been great interest in within-host adaptations [85,86]. For example, a genomic study revealed how a zoonotic chronic infection of an immunocompromised patient by *Bordetella hinzii* resulted in rapid genomic adaptation [87]. Inactivation of DNA proofreading activity in combination with oxidative attack and rapid metabolic adaptation were key events. Another very interesting comparative study revealed how different bacterial pathogens share some common adaptive strategies during within-host chronic infections. These include the mutation of flagellar genes, shifts from siderophore-based to heme-based iron scavenging, virulence attenuation, and adaptations in glycerol-phosphate metabolism [88].

Comparative analyses of core genomes/proteomes between evolutionarily related groups may also reveal certain adaptations towards pathogenicity. For example, a comparative analysis of various *P. aeruginosa* strains against other *Pseudomonas* groups highlighted the presence of several *P. aeruginosa* core-specific genes that are involved in its pathogenicity such as metal-scavenging, motility, mucin production, toxin–antitoxin systems, and membrane remodelling during stress [89]. Genome wide association studies (GWAS) are also being performed in pathogenic bacteria, in order to identify traits related to pathogenesis such as antibiotic susceptibility or host specificity [90].

## 9. The Contribution of Bacterial Pathogen Genomics in Forensics, Epidemiology, and Food Safety

From a practical point of view, WGS has already transformed the forensic analysis of outbreaks of bacterial disease because it allows for the rapid detection of pathogens and high-resolution phylogenomic analysis of their relationships and how they spread [12,91]. One very famous early investigation that constituted proof-of-concept for forensics was the Amerithrax incident in 2001 and its subsequent forensic genomic study [92,93]. In that landmark investigation, whole genome sequencing and comparative genomics were employed in order to develop high-resolution genetic markers that constituted a unique fingerprint for the Anthrax spores that were used in the 2001 letter-attacks. The markers were later used in order to investigate the various samples collected by the FBI, and thus helped to identify the source of the spores. Another key example is the 2010 Haitian cholera outbreak that was determined by genomics and phylogenomics to have been caused by Nepalese UN soldiers [94]. A third notable example is the genomic and phylogenomic analysis of methicillin-resistant *S. aureus* isolates from different geographic regions and from a hospital outbreak [95]. This analysis demonstrated the superior resolution of WGS over conventional MLST analysis in delineating microevolutionary relationships, and revealed the global geographic structure of that lineage and the ability to trace person-to-person transmission within a hospital environment. Another very interesting finding of that study was the observation of homoplasic SNPs in drug-resistance genes. This was a clear indication that clinical practice is a major driver of pathogen evolution.

Concerning food safety, the investigation of an outbreak of Shiga-toxin-producing *E. coli* in Germany between May and June 2011, with more than 3000 people infected, demonstrated the advantages of WGS [96]. Nowadays, GenomeTrakr is a large network of U.S. federal, state, university, and hospital labs that is utilising WGS in order to detect and analyse outbreaks of food-borne illness caused by pathogens [97]. Although the annual investment is around USD 22 million, the annual health benefits are estimated to be at nearly USD 500 million [98]. Furthermore, the European Food Safety Authority (EFSA) has also adopted WGS for the bacterial strain taxonomic identification and characterisation of potential traits of concern [99]. A retrospective investigation of two outbreaks of food-borne disease due to *E. coli* (in 2012 and 2013) clearly demonstrated that the use of WGS is far superior to the conventional approaches for the generation of information on virulence, AMR genotypes, and accurate cluster identification [100].

## 10. Clinical Importance of Bacterial Pathogen Genomics

Recently, with the significant decrease in sequencing costs with short-read technologies and the introduction of more reliable long-read technologies, the field of clinical microbiology is undergoing a transformation [12]. For example, reference laboratories of Public Health England and the Scottish Healthcare Associated Infection Prevention Institute have adopted WGS as a routine method to analyse samples from bacterial pathogens such as *Salmonella*, *E. coli* and *Shigella*, *Listeria*, and *Campylobacter* [101]. The WGS studies that exploited long-read sequencing not only determined the nosocomial transmission and AMR profiles, but also delineated the role of plasmid spread [25]. WGS has been evaluated against traditional phenotypic approaches, in order to determine AMR profiles with mixed or even poor results initially [102]. However, more recent studies have now demonstrated very high levels of concordance [28,55,103].

Technical challenges to the more routine use of these approaches in a clinical setting do, however, remain to be overcome. Several recent studies have been performed in order to evaluate the various protocols, sequencing platforms, and bioinformatics pipelines concerning clinical microbiology and epidemiology. However, mixed results have been observed in terms of the reproducibility and concordance among different laboratories. For example, a multi-centre ring trial of *S. aureus* involving nine Swiss laboratories revealed that differential sample preparation and SNP calling procedures led to different sets of informative SNPs for cluster identification, although the phylogenetic trees and cluster identification were highly reproducible [104]. Another multi-centre study of nine laboratories focused on assessing the various bioinformatics pipelines for predicting AMR by providing every centre with the same set of short-read WGS data (Illumina NextSeq and HiSeq) from the clinical isolates. Again, some discordance was observed in predicting the antimicrobial susceptibility [105]. Along the same lines, a multi-centre study involving 13 major Dutch health care-affiliated centres revealed that, even when analysing the same raw sequencing data (Illumina), there were discrepancies in reporting the antimicrobial resistance, multi-locus sequence typing (MLST), and outbreak clusters [106]. Importantly, a clinical study of bile-duct cultures from pancreatic head resections compared the efficiency, cost, and time needed to obtain actionable results in terms of surgical site infection and antibiotic stewardship from the classical aerobic/anaerobic cultures and Oxford Nanopore sequencing [107]. Nanopore sequencing identified more microbes per positive sample, was faster (8 vs. 98 h), but was costlier (USD 165 vs. USD 38). Different library preparation protocols for Illumina sequencing also have an impact [108]. Experts in the clinical field have highlighted the need for such WGS-based approaches to be of high quality and to produce clinically actionable results within a useful time-frame that are also clear and meaningful [101].

## 11. The Shape of Things to Come

Advances in sequencing technologies and computational pipelines have enabled pathogen genomics to come a very long way in a relatively short time. As with any rapidly progressing field of research, challenges and opportunities arise that need to be addressed on the road to reaching their full potential. Technological breakthroughs will determine the pace at which bacterial pathogen genomics will transform the fields of forensics, food-safety, and clinical microbiology in both developed and developing countries. The technologies of long-read sequencing are rapidly improving their durability and base-calling accuracy to a level that will soon be comparable to that of short-read technologies. Whether it will be PacBio, Oxford Nanopore, or some other new technology such as solid-state nanopores [109] remain to be seen. However, once such a milestone is reached, it is reasonable to assume that metagenome-assembled genomes (MAGs) will become the standard approach.

A very important goal for the clinical and food-safety settings is to progress from the WGS of cultured bacterial isolates towards faster, simpler, mobile/on-site culture-independent metagenomic analyses of samples, while retaining the ability to detect all the relevant bacterial genomes, their abundance, and their properties. In order to obtain all this information, 16S rRNA metagenomics is not sufficient, but complete or even draft genomes from metagenomics (metagenomic-assembled genomes—MAGs) are needed. This has the clear advantage of being able to assess the bacterial communities in an unbiased way, and several studies have proven the feasibility of this approach [110,111,112]. However, given the species-complexity of the uncultured samples, at the moment, it is challenging to obtain high-quality MAGs due to the gaps, assembly errors, chimeras, within-population diversity, and contamination [113]. A recent comparative study of HiSeq-only, Minion-only, and a hybrid approach demonstrated that the last produced the best results [114]. Similarly, another recent study adopted a hybrid approach of short- and long-(Nanopore) read sequencing and produced large numbers (over 1000) of high-quality MAGs from complex microbial communities (of Danish wastewater treatment plants) [115]. A number of complex bioinformatics pipelines are currently being applied and developed to analyse such challenging data (for comprehensive reviews see [116,117]). Cell-free plasma Next-Generation Sequencing (cfNGS), a form of plasma metagenomics, is already being adopted with success in certain clinical settings such as paediatric complicated pneumonia [118].

An additional challenge is to make the sequencing technologies more mobile, thus enabling the collection and processing of data in remote environments or places where resources may be limited or sample transferring is complicated. The recent use of ONT devices in studying the antimicrobial resistance and phylogeny of *Neisseria gonorrhoeae* clinical isolates in Kenya indicates useful progress in this direction [119]. For time-critical applications such as those aimed at infection surveillance and tracking to prevent and control the spread of disease, or the determination of virulence serotypes or antimicrobial resistance genotypes in a clinical setting, the speed of generating actionable results from samples taken is vital. This is a research space that the third-generation sequencing platforms are targeting, leveraging the speed and simplicity of their sample preparation steps and their ability to produce the sequence in real-time. However, sequencing data processing and computational analysis in a timely manner is also part of the equation. Generating easy-to-understand reports for clinicians or non-experts to interpret and appropriately action is also important and is an area that will need careful development in the future.

Another important aspect will be to properly sample the entire bacterial biodiversity in terms of the complete genomes, plasmids, and gene families. Such a goal is very important in order to obtain a deeper understanding of how bacteria and their pathogenicity evolve. In addition, a broader sampling of the existing gene/protein families and their diversity is very important in order to detect and prevent any intentional misuse of synthetic biology [120].

Finally, despite the tsunami of genomic data that are expected to be produced in the near future, storing all of these data into large repositories will not be sufficient. Species-specific databases of bacterial pathogens of high importance will need to be further developed and continuously maintained by dedicated expert annotators who understand the nature and molecular biology of each particular organism [121]. High-throughput genomics will also need to co-exist and be co-funded with low-throughput focused experimental biochemistry and molecular biology. What is the use of a genome, if a large proportion of its genes are of unknown function?

## Data Availability

Not applicable.
